# Stroke in young adults, stroke types and risk factors: a case control study

**DOI:** 10.1186/s12883-022-02853-5

**Published:** 2022-09-06

**Authors:** Priscilla Namaganda, Jane Nakibuuka, Mark Kaddumukasa, Elly Katabira

**Affiliations:** 1 Kiruddu National Referral Hospital, P.O. Box 6553, Kampala, Uganda; 2 Mulago National Referral Hospital, Mulago Hospital Complex, P.O. Box 7272, Kampala, Uganda; 3 Department of Medicine, School of Medicine, College of Health Sciences, Makerere University, Kampala, Uganda; 4 Infectious Diseases Institute, Makerere University, Kampala, Uganda

**Keywords:** Young adults, Stroke, Risk factors

## Abstract

**Background:**

Stroke is the second leading cause of death above the age of 60 years, and the fifth leading cause in people aged 15 to 59 years old as reported by the World Health Organization global burden of diseases. Stroke in the young is particularly tragic because of the potential to create long-term disability, burden on the victims, their families, and the community at large. Despite this, there is limited data on stroke in young adults, and its risk factors in Uganda. Therefore, we determined the frequency and risk factors for stroke among young adults at Mulago hospital.

**Methods:**

A case control study was conducted among patients presenting consecutively to the general medical wards with stroke during the study period September 2015 to March 2016. A brain Computerized Tomography scan was performed to confirm stroke and classify the stroke subtype. Controls were patients that presented to the surgical outpatient clinic with minor surgical conditions, matched for age and sex. Social demographic, clinical and laboratory characteristics were assessed for both cases and controls. Descriptive statistics including frequencies, percentages, means, and standard deviation were used to describe the social demographics of case and controls as well as the stroke types for cases. To determine risk factors for stroke, a conditional logistic regression, which accounts for matching (e.g., age and sex), was applied. Odds ratio (with 95% confidence interval) was used as a measure for associations.

**Results:**

Among 51 patients with stroke, 39(76.5%) had ischemic stroke and 12(23.5%) had hemorrhagic stroke. The mean age was 36.8 years (SD 7.4) for stroke patients (cases) and 36.8 years (SD 6.9) for controls. Female patients predominated in both groups 56.9% in cases and 52.9% in controls. Risk factors noted were HIV infection, OR 3.57 (95% CI 1.16–10.96), elevated waist to hip ratio, OR 11.59(95% CI 1.98–68.24) and sickle cell disease, OR 4.68 (95% CI 1.11–19.70). This study found a protective effect of oral contraceptive use for stroke OR 0.27 95% CI 0.08–0.87. There was no association between stroke and hypertension, diabetes, and hyperlipidemia.

**Conclusion:**

Among young adults with stroke, ischemic stroke predominated over hemorrhagic stroke. Risk factors for stroke were HIV infection, elevated waist to hip ratio and sickle cell disease.

## Background

Stroke is the second leading cause of death above the age of 60 years, and the fifth leading cause in people aged 15 to 59 years old as reported by the World Health Organization (WHO) global burden of diseases [1]. The severity of stroke in the young is relatively low in developed countries ranging from 2 -7% in Italy and USA respectively [2, 3]. In Africa, on the other hand the prevalence of stroke among young adults is 12.9% in Nigeria [4], 31% in South Africa [5], 28.9% in Morocco [6]. The incidence of ischemic stroke in the young has been increasing globally over the last 2–3 decades. From the Danish National Patient Register, the incidence rates of first‐time hospitalizations for ischemic stroke and transient ischemic attack (TIA) in young adults have increased substantially since the mid 1990s while the incidences of hospitalizations for intracerebral hemorrhage and subarachnoid hemorrhage remained stable during the study period [7].

In Uganda, literature on stroke in young adults is limited however results of a study done among acute stroke patients admitted to the national referral hospital (Mulago hospital) showed a 30-day mortality of 43.8%. Out of 133 patients, 32 patients (25%) were less than 51 years old. Out of the 56 patients that died, 13 patients (23%) were less than 51 years [8].

Rapid western cultural adaption (sedentary lifestyle, deleterious health behavior like consumption of tobacco and alcohol and high fat/cholesterol diet) and Human immunodeficiency syndrome/ Acquired immunodeficiency syndrome (HIV/AIDS) that is highly prevalent in Africa has accelerated risk factors and increased the burden of stroke [9].

Most literature indicates that the traditional risk factors i.e., hypertension, diabetes mellitus and dyslipidemia are still the commonest risk factors with hypertension having the highest frequency. Other risk factors common to the young include smoking, excessive alcohol intake, illicit drug use, oral contraceptive use and migraine [10].

Although stroke is predominantly a disease of the middle age and the elderly, its occurrence in younger age groups is not rare. Stroke in young adults seems to be increasing and is particularly tragic because of the potential to create long-term disability, burden on the victims, their families, and the community at large such as Uganda. Despite the huge socioeconomic impact of stroke in this age group, there is a scarcity of data regarding stroke in young adults in sub-Saharan Africa including Uganda. Effective stroke prevention strategies in the young require comprehensive information on risk factors and possible causes. Although case reports and etiologic investigations of possible causes of stroke in the young have been identified especially in developed countries, there is limited data on risk factors in Africa Uganda inclusive. Information obtained from this study will fill the knowledge gap in this area of stroke in the young which will inform institutional strategies on prevention and management of stroke in this age group. This study, therefore, seeks to determine the frequency of stroke types and risk factors for this population.

The aims of the study were:

1. To determine the frequency of stroke types among young adults on the general medical wards in Mulago hospital between September 2015 and March 2016.
2. To determine the risk factors for stroke (i.e., ischemic, and hemorrhagic stroke) among young adults on the general medical wards in Mulago hospital between September and March 2016.

## Methods

This was a case control study. Cases were defined as patients with a confirmed diagnosis of stroke by brain computerized tomography (CT) scan that met the inclusion criteria. Controls were defined as patients with minor surgical conditions that met the inclusion criteria. The study was carried out in Mulago hospital which is the national referral hospital in Uganda as well as the teaching hospital of Makerere University College of health sciences. It has a bed capacity of 1500 beds and has both inpatient wards, outpatient departments both for medical and surgical specialties. It has a radiological department with CT scan and highly trained personnel and a well-equipped laboratory. Cases were recruited consecutively from the medical wards specifically on the neurology ward of Mulago hospital. Patients on the neurology ward are managed by physicians that have had additional training in the management of neurological conditions.

Controls were recruited from general surgical outpatient departments from Mulago hospital. They were matched for age and sex. Eligible patients were patients aged 15–45 years, confirmed diagnosis of stroke on brain CT scan and with a written informed consent or assent for patients less than 18 years. These included patients with intracranial hemorrhages and ischemic stroke, none had subarachnoid hemorrhage. Patients were excluded if they were unconscious and with no valid surrogate (next of kin) and HIV positive with opportunistic infections. Patients eligible as control were, patient aged 15–45 years, minor surgical condition, written informed consent or assent for patients less than 18 years. Patients with features of stroke secondary to non-vascular causes like trauma, tumors were excluded as controls. For controls, we chose patients with minor surgical conditions because we wanted controls to be hospital patients but with non-medical conditions that could confound our findings. Such conditions included lacerations, hernias, lipomas, ingrown toenails, circumcision.

Based on the catchment area of Mulago, patients with minor surgical conditions are likely to have similar social economic status and come from similar neighborhoods as would health controls living in the catchment areas as patients with stroke.

The best alternative would have been healthy controls from the neighborhoods of the patients with stroke, but this would have been resource consuming.

The sample size was calculated assuming a prevalence of 62.2% of hypertension among the stroke patients as was indicated in a similar study among the young Thai adults in Bangkok, Thailand (Bandasak et al., 2011) [11]. We also assumed that the risk for stroke is higher among the hypertensive with an OR of 3. With this sample size, we were powered to detect associations with other risk factors like smoking (OR 2.6) [12], diabetes (OR 13.2 for black men and 22.1 for black women) [13].

With these assumptions, a sample size of 51 cases and 51 controls was found sufficient with 80% power and 0.05 level of significance.

### Sampling procedure

All young patients admitted on the general medical wards suspected of having stroke were screened and brain CT scan done. Once a diagnosis of stroke was confirmed on CT scan, participants who consented to participate in the study were recruited consecutively, a standardized questionnaire administered by the research team for those patients able to communicate. For patients not able to communicate, consent and information were obtained through the care givers. Controls were selected from the general surgical outpatient clinic using consecutive sampling method. This was done after we had obtained all the cases. These were matched for age and sex until the sample size was accrued.

Information was collected on:

1. Social demographic characteristics i.e., age, sex, level of education, occupation, religion, history of smoking and alcohol consumption, history of illicit drug use, history of oral contraceptive use.
2. Clinical examination included general physical examination, blood pressure using a digital blood pressure machine. For patients who were too weak to sit up, blood pressure measurement was taken in supine position. For those able to sit, it was taken in the sitting position. The two blood pressure measurements were taken at an interval of 5 min and the average blood pressure recorded as the final blood pressure.
  Physical measurements for the weight and hip were taken using a stretchable tape measure. Waist measurements were taken at the narrowest point-umbilicus and hip measurements at the widest point- buttocks. A waist to hip ratio was obtained and recorded on the questionnaire.
3. Blood was drawn for laboratory tests; high density lipoprotein, low density lipoprotein (HDL/LDL), fasting blood sugar, full blood count, Hb electrophoresis, prothrombin time/ international normalization ratio (PT/INR), HIV serology, Treponema pallidum hemagglutination (TPHA).
4. Other information obtained was history and family history of diabetes and hypertension.
  The general surgical outpatient clinic runs every Tuesday, and Thursday in Old Mulago hospital Participants were identified at the surgical outpatient clinic. Those matching the age and sex of the cases were recruited, written consent/assent obtained, and questionnaire was administered by the PI. The procedure as explained above was followed for the controls.

### Data collection

A pre-tested and standardized questionnaire was used as a data collection tool. The principal investigator administered the questionnaire to the participants in data collection. Data on socio demographics and past medical history was collected.

Results from imaging and laboratory investigations were also recorded into the questionnaire.

Data collected was double entered into the computer using EPI-DATA (version 3.1) software to minimize data entry errors. Data was then backed up and archived in both soft and hard copy to avoid losses. Confidentiality was ensured using code numbers instead of patients’ names. Questionnaires were stored in a lockable cabinet for safety.

### Data analysis

Data was analyzed using STATA Version 12 (StataCorp. 2011. *Stata Statistical Software: Release 12*. College Station, TX: StataCorp LP). Descriptive statistics were used to describe characteristics of the study participants and the stroke subtypes which included frequencies, percentages, means and standard deviation. To determine factors associated with stroke, a conditional logistic regression, which accounts for matching (e.g., age and sex), was applied. Odds ratio (with 95% confidence interval) was used as a measure for associations. Factors with *p*-values < 0.2 at a bi-variable analysis were entered into a multiple conditional logistic regression to obtain the adjusted estimates. Factors whose 95% confidence interval for the odds ratio that excludes a 1 or whose *p*-value < 0.05, were considered statistically significant at the adjusted level. Post-hoc power calculation was performed for the adjusted analysis to check if there was enough power to detect a difference between cases and controls.

### Quality control

To ensure quality of results several measures were undertaken, these included:

- The questionnaires were pre-tested and standardized before study commenced.
- The research team administered the structured, pre- coded and pre-tested questionnaire to enrolled participants on a face-to-face basis and brain CT scans were done by competent and well-trained radiology technicians and interpretation done by a specialist radiologist at the Radiology Department of Mulago hospital.
- The questionnaires were checked for completeness at the end of every interview. The two files were compared, and any discordance corrected against data recorded with the questionnaire. The data were then backed up.

### Ethical consideration

Written informed consent/ assent was obtained from all participants or their parent/guardian or legal authorized representative to participate in the study. Ethical approval was obtained from Makerere University, school of medicine research and ethics committee (SOMREC) (reference number #REC REF 2015–105).

Confidentiality was ensured using code numbers instead of patients’ names. Questionnaires were stored in a lockable cabinet for safety.

## Results

### Profile of the study

**Fig. 1:**
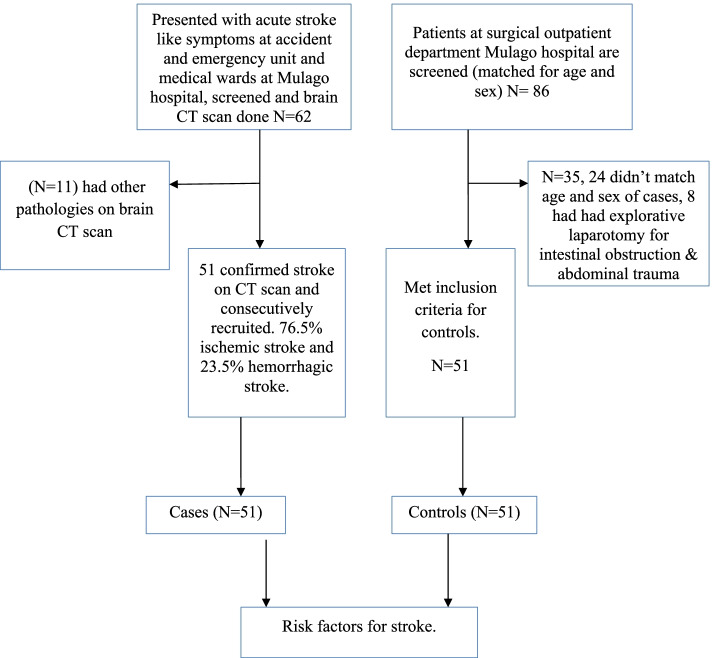
Patient flow diagram

Enrollment of study participants was carried out between September 2015 to March 2016 in Mulago hospital. The patient flow diagram for cases and controls is as shown in Fig. 1.

### Social demographic characteristics of the study population

A total of 51 cases aged 18 to 45 years and the same number of hospital control matched for age and sex were identified. The mean age of cases was 36.8 years (standard deviation (SD) 7.4) and the control was 36.8 years (SD 6.9). Females predominated in both groups with 56.9% in cases and 52.9% in controls. There was no significant difference in other baseline characteristics between cases and controls except in oral contraceptive use, waist to hip ratio, HIV status and sickle cell disease. Details of the social demographic characteristics are shown in Table 1.

**Table 1:** Social Demographic characteristics of study participants

	**Cases (stroke)** **(*N* = 51)**	**Controls** **(*N* = 51)**	***P* value**
**Age in years:** Mean (SD)	36.8 (7.4)	36.8 (6.9)	
	**n (%)**	**n (%)**	
**Categories of age in years**			
18–25	5 (9.8)	4 (7.8)	0.675
26–35	13 (25.5)	17 (33.3)	
36–45	33 (64.7)	30 (58.8)	
**Sex**			
Male	22 (43.1)	24 (47.1)	0.691
Female	29 (56.9)	27 (52.9)	
**Religion**			
Protestant	23 (45.1)	14 (27.5)	0.051
Catholic	14 (27.5)	17 (33.3)	
Moslem	10 (19.6)	7 (13.7)	
Other	4 (7.8)	13 (25.5)	
**Marital status**			
Married	32 (62.8)	31 (60.8)	0.880
Never married (single)	10 (19.6)	12 (23.5)	
Married before	9 (17.7)	8 (15.7)	
**Highest level of education**			
Primary	22 (43.1)	19 (37.3)	0.642
Secondary	16 (31.4)	21 (41.2)	
Tertiary	5 (9.8)	6 (11.8)	
**Current smoking**			
Yes	1 (2.0)	2 (3.9)	> 0.999
No	50 (98.0)	49 (96.1)	
**Former smoker**			
Yes	4 (7.8)	5 (9.8)	> 0.999
No	47 (92.2)	46 (90.2)	
**Current alcohol consumption**			
Yes	11 (21.6)	11 (21.6)	> 0.999
No	40 (78.4)	40 (78.4)	
**Illicit drug use**			
Yes	1 (2.0)	1 (2.0)	> 0.999
No	50 (98.0)	50 (98.0)	
**Oral contraceptive use**			
Yes	7 (24.1)	14 (51.9)	**0.047**
No	22 (75.9)	13 (48.1)	

### Clinical characteristics of the study participants

The mean fasting blood sugar was 6.6 (SD 3.9) for cases and 5.3 (SD 0.7) for controls. This was statistically significant with a *p* value of 0.015. Waist to hip ratio was also statistically significant with a *p* value of 0.007. Cases with an elevated wait to hip ratio were 14 (27.5%) and controls were 3 (5.9%). Table 2 shows the baseline clinical characteristics of the study participants.

**Table 2:** Clinical characteristics of the study participants

	**Cases (stroke** ***N* = 51**	**Controls** ***N* = 51**	***P* value**
	**Mean (SD)**	**Mean (SD)**	
Systolic blood pressure	134.3 (31.9)	129.0 (22.3)	0.339
Diastolic blood pressure	85.0 (22.4)	80.5 (18.7)	0.274
Fasting blood sugar	6.6 (3.9)	5.3 (0.7)	**0.015**
	**n (%)**	**n (%)**	***P* value**
**Hypertensive**			
Yes	21 (41.2)	13 (25.5)	0.093
No	30 (58.8)	38 (74.5)	
**Status of fasting blood sugar**			
High	6 (12.0)	1 (2.0)	0.060
Normal	44 (88.0)	50 (98.0)	
**History of hypertension**			
Yes	14 (27.5)	8 (15.7)	0.149
No	37 (72.6)	43 (84.3)	
**History of family hypertension**			
Yes	16 (31.4)	18 (35.3)	0.674
No	35 (68.6)	33 (64.7)	
**History of diabetes**			
Yes	2 (4.8)	0 (0.0)	0.495
No	49 (96.1)	51 (100.0)	
**History of family diabetes**			
Yes	4 (7.8)	8 (15.7)	0.357
No	47 (92.2)	43 (84.3)	
**History of heart disease**			
Yes	2 (3.9)	0 (0.0)	0.495
No	49 (96.1)	51 (100.0)	
**Waist to hip ratio**			
High	14 (27.5)	3 (5.9)	**0.007**
Normal	37 (72.6)	48 (94.1)	

### Laboratory characteristics of the study participants

HIV serology and Hb electrophoresis were statistically significant with a *p* value of 0.076 and 0.023 respectively. 18 patients (35.3%) were reactive for HIV among cases and controls 10 (19.6%). 12 patients (23.5%) had abnormal Hb electrophoresis among cases controls 3 (5.9%). Table 3 shows the laboratory characteristics of the study participants.

**Table 3:** Laboratory characteristics of the study participants

	**Cases (strokes)** **(*N* = 51)**	**Controls** **(*N* = 52)**	***P* values**
**Hemoglobin**			
Normal	40 (78.4)	44 (86.3)	0.299
Low/high	11 (21.6)	7 (13.7)	
**Platelet count**			
Normal	42 (82.4)	43 (84.3)	0.790
Low/high	9 (17.7)	8 (15.7)	
**Leucocyte count**			
Normal	44 (86.3)	48 (94.1)	0.318
Low/high	7 (13.7)	3 (5.9)	
**LDL**			
Normal	37 (72.6)	38 (74.5)	0.822
Low/high	14 (27.5)	13 (25.5)	
**HDL**			
Normal	29 (56.9)	30 (58.8)	0.841
Low/high	22 (43.1)	21 (41.2)	
**INR**			
Normal	37 (72.6)	40 (78.4)	0.490
Low/high	14 (27.5)	11 (21.6)	
**Prothrombin time (PT)**			
Normal	38 (74.5)	40 (78.4)	0.641
Low/high	13 (25.5)	11 (21.6)	
**TPHA**			
Reactive	5 (9.8)	5 (9.8)	> 0.999
Non-reactive	46 (90.2)	46 (90.2)	
**HIV serology**			
Non-Reactive	33 (64.7)	41 (80.4)	**0.076**
Reactive	18 (35.3)	10 (19.6)	
**HB electrophoresis**			
AA	39 (76.5)	48 (94.1)	**0.023**
SS	12 (23.5)	3 (5.9)	

### Stroke types

#### Stroke types by social demographic characteristics of cases

Among 62 patients, who had brain CT scan done, 11 patients had non stroke pathologies (4 had brain abscesses, 7 patients had ring enhancing lesions suggestive of toxoplasmosis). Among 51 patients with stroke confirmed on CT scan, the frequency of ischemic stroke was 76.5% and hemorrhagic stroke was 23.5%.

Most participants with ischemic or hemorrhagic stroke were in the age group 36–45 years. Females predominated in both ischemic and hemorrhagic stroke. Details of the social demographic characteristics by stroke types are shown in Table 4.

**Table 4:** Social demographic characteristics by stroke types

**Demographic characteristics (*N* = 51)**	**Ischemic** **N (%)**	**Hemorrhagic** **N (%)**	***P* values**
Overall	39 (76.5)	12 (23.5)	
**Age in years**			
18–25	4 (10.3)	1 (8.3)	> 0.999
26–35	10 (25.6)	3 (25.0)	
36–45	25 (64.1)	8 (66.7)	
**Sex**			
Male	17 (43.6)	5 (41.7)	0.906
Female	22 (56.4)	7 (58.3)	
**Religion**			
Protestant	18 (46.2)	5 (41.7)	0.222
Catholic	8 (20.5)	6 (50.0)	
Moslem	9 (23.1)	1 (8.3)	
Other	4 (10.3)	0 (0.0)	
**Marital status**			
Married	23 (59.0)	9 (75.0)	0.721
Never married (single)	8 (20.5)	2 (16.7)	
Married before	8 (20.5)	1 (8.3)	
**Highest level of education**			
Primary	17 (43.6)	5 (41.7)	0.887
Secondary	11 (28.2)	5 (41.7)	
Tertiary	4 (10.3)	1 (8.3)	
Never attended school	7 (18.0)	1 (8.3)	

### Clinical and laboratory characteristics by stroke types

Majority of patients with hemorrhagic stroke were hypertensive (91.7%) compared to only 25.6% among patients with ischemic stroke. Details of the clinical and laboratory characteristics of the study participants by stroke subtypes are shown in Table 5.

**Table 5:** Shows the clinical and laboratory characteristics by stroke types

**Clinical characteristics (*N* = 51)**	**Ischemic (39)** **N (%)**	**Hemorrhagic (*N* = 12)** **N (%)**	***P* values**
Hypertensive (Yes)	10 (25.6)	11 (91.7)	> 0.001
**Status of fasting blood sugar**			
High	4(10.5)	2(16.7)	0.621
Normal	34(89.5)	10(83.3)	
**Waist to hip ratio**			
High	13(33.3)	1(8.33)	0.142
Normal	26(66.7)	11(91.7)	
**Current smoking (Yes)**	1(2.56)	0(0.00)	> 0.999
**Current alcohol consumption (Yes)**	8(20.5)	3(25.0)	0.706
**Oral contraceptive use (Yes)**	3(13.0)	4(57.4)	0.033
**LDL**			
Normal	29(74.4)	8(72.6)	0.715
Low/high	10(25.6)	4(33.3)	
**HDL**			
Normal	23(59.0)	6(50.0)	0.583
Low/high	16(41.0)	6(50.0)	
**Prothrombin time (PT)**			
Normal	29 (74.7)	9(75.0)	> 0.999
Low/high	10(25.6)	3(25.0)	
**HIV serology**			
Reactive	16(41.0)	2(16.7)	0.174
Non-reactive	23(59.0)	10(83.3)	
**HB electrophoresis**			
AA	30(77.0)	9(75.0)	> 0.999
SS	9(23.1)	3(25.0)	

### Risk factors for stroke at univariate analysis

#### Social demographic characteristics at univariate analysis

Oral contraceptive use showed a significant difference with an unadjusted OR of 0.27 (95% CI 0.08–0.87) case subjects 23.3% and control subjects 56.5%. Belonging to other religion (seventh day advent, Pentecostal) was statistically significant with a *p* value of 0.009, OR 0.17. These findings are detailed in Table 6 below.

**Table 6:** Social demographic characteristics at univariate analysis

	**Cases (stroke)** **(*N* = 51)**	**Controls** **(*N* = 51)**	**Odds Ratio**^a^ **95 (% CI)**	***P* value**
**Age in years**^b^**:** Mean (SD)	36.8 (7.4)	36.8 (6.9)		
	**n (%)**	**n (%)**		
**Categories of age in years**				
18–25	5 (9.8)	4 (7.8)		
26–35	13 (25.5)	17 (33.3)		
36–45	33 (64.7)	30 (58.8)		
**Sex**^b^				
Male	22 (43.1)	24 (47.1)		
Female	29 (56.9)	27 (52.9)		
**Religion**				
Protestant	23 (45.1)	14 (27.5)	Reference	
Catholic	14 (27.5)	17 (33.3)	0.44 (0.16 – 1.20)	0.110
Moslem	10 (19.6)	7 (13.7)	0.71 (0.22 – 2.28)	0.567
Other	4 (7.8)	13 (25.5)	0.17 (0.05 – 0.65)	**0.009**
**Marital status**				
Married	32 (62.8)	31 (60.8)	Reference	
Never married (single)	10 (19.6)	12 (23.5)	0.70 (0.24 – 2.02)	0.509
Married before	9 (17.7)	8 (15.7)	0.88 (0.27 – 2.79)	0.823
**Highest level of education**				
Primary	22 (43.1)	19 (37.3)	Reference	
Secondary	16 (31.4)	21 (41.2)	0.58 (0.23 – 1.47)	0.250
Tertiary	5 (9.8)	6 (11.8)	0.62 (0.16 – 2.38)	0.485
**Current smoking**				
Yes	1 (2.0)	2 (3.9)	0.34 (0.03 – 3.95)	0.390
No	50 (98.0)	49 (96.1)	Reference	
**Former smoker**				
Yes	4 (7.8)	5 (9.8)	0.80 (0.19 – 3.30)	0.755
No	47 (92.2)	46 (90.2)	Reference	
**Current alcohol consumption**				
Yes	11 (21.6)	11 (21.6)	0.97 (0.38 – 2.48)	0.946
No	40 (78.4)	40 (78.4)	Reference	
**Illicit drug use**				
Yes	1 (2.0)	1 (2.0)	1.31 (0.08 – 21.07)	0.849
No	50 (98.0)	50 (98.0)	Reference	
**Oral contraceptive use**				
Yes	7 (24.1)	14 (51.9)	0.30 (0.09–0.98)^c^	**0.047**
No	22 (75.9)	13 (48.1)	Reference	

^a^ Obtained accounting for matching by age and sex using a conditional logistic regression

^b^ No comparison made because matching was done using these variables (age and sex)

^c^ Accounting for matching was done only for age because contraceptive use applies only to female gender

### Clinical characteristics at univariate analysis

There was a significant difference in waist to hip ratio between cases (27.5%) and controls (5.9%), with unadjusted OR 6.85 (CI 1.70–27.62). HIV serology with an unadjusted OR of 2.64 (95% CI 1.03–6.82). Hb electrophoresis with an unadjusted OR of 4.31 (95% CI- 1.15–16.17). Fasting blood sugar with an unadjusted OR of 1.64 (95% CI 1.02–2.62). Details of the above findings are shown in Table 7 below.

**Table 7:** Clinical characteristics of study participants at univariate analysis

	**Cases (stroke)** ***N* = 51**	**Controls** ***N* = 51**		
	**Mean (SD)**	**Mean (SD)**	**Odds Ratio**^a^ **95% CI**	***P*** **value**
Systolic blood pressure	134.3 (31.9)	129.0 (22.3)	1.01 (0.99 – 1.02)	0.397
Diastolic blood pressure	85.0 (22.4)	80.5 (18.7)	1.01 (0.99 – 1.03)	0.319
Fasting blood sugar	6.6 (3.9)	5.3 (0.7)	1.64 (1.02 – 2.62)	**0.040**
	**n (%)**	**n (%)**	**Odds Ratio**^a^ **95% CI**	***P* value**
**Hypertensive**				
Yes	21 (41.2)	13 (25.5)	1.78 (0.77 – 4.12)	0.175
No	30 (58.8)	38 (74.5)	Reference	
**Status of fasting blood sugar**				
High	6 (12.0)	1 (2.0)	5.61 (0.12 – 48.65)	0.118
Normal	44 (88.0)	50 (98.0)	Reference	
**History of hypertension**				
Yes	14 (27.5)	8 (15.7)	1.77 (0.66 – 4.75)	0.257
No	37 (72.6)	43 (84.3)	Reference	
**History of family hypertension**				
Yes	16 (31.4)	18 (35.3)	0.77 (0.34 – 1.75)	0.537
No	35 (68.6)	33 (64.7)	Reference	
**History of diabetes**				
Yes	2 (4.8)	0 (0.0)	3.12 (0.24 – 167.1) ^b^	0.308
No	49 (96.1)	51 (100.0)	Reference	
**History of family diabetes**				
Yes	4 (7.8)	8 (15.7)	0.39 (0.10 – 1.44)	0.156
No	47 (92.2)	43 (84.3)	Reference	
**History of heart disease**				
Yes	2 (3.9)	0 (0.0)	3.12 (0.24 – 167.1) ^b^	0.308
No	49 (96.1)	51 (100.0)	Reference	
**Waist to hip ratio**^c^				
High	14 (27.5)	3 (5.9)	6.85 (1.70 – 27.62)	**0.007**
**Hemoglobin**				
Normal	40 (78.4)	44 (86.3)	Reference	
Low/high	11 (21.6)	7 (13.7)	1.63 (0.56 – 4.73)	0.369
**Platelet count**				
Normal	42 (82.4)	43 (84.3)	Reference	
Low/high	9 (17.7)	8 (15.7)	1.17 (0.40 – 3.37)	0.777
**Leucocyte count**				
Normal	44 (86.3)	48 (94.1)	Reference	
Low/high	7 (13.7)	3 (5.9)	2.58 (0.63 – 10.59)	0.188
**LDL**				
Normal	37 (72.6)	38 (74.5)	Reference	
Low/high	14 (27.5)	13 (25.5)	1.07 (0.43 – 2.63)	0.891
**HDL**				
Normal	29 (56.9)	30 (58.8)	Reference	
Low/high	22 (43.1)	21 (41.2)	0.97 (0.43 – 2.16)	0.933
**INR**				
Normal	37 (72.6)	40 (78.4)	Reference	
Low/high	14 (27.5)	11 (21.6)	1.41 (0.58 – 3.46)	0.450
**Prothrombin time (PT)**				
Normal	38 (74.5)	40 (78.4)	Reference	
Low/high	13 (25.5)	11 (21.6)	1.32 (0.53 – 3.29)	0.556
**TPHA**				
Reactive	5 (9.8)	5 (9.8)	Reference	
Non-reactive	46 (90.2)	46 (90.2)	1.04 (0.28 – 3.93)	0.953
**HIV serology**				
Non-Reactive	33 (64.7)	41 (80.4)	Reference	
Reactive	18 (35.3)	10 (19.6)	2.64 (1.03–6.82)	**0.044**
**HB electrophoresis**				
AA	39 (76.5)	48 (94.1)	Reference	
SS	12 (23.5)	3 (5.9)	4.31 (1.15 – 16.17)	**0.030**

^a^ Obtained accounting for matching by age and sex using a conditional logistic regression

^b^ Obtained by adding a 1 on each cell count (due to zero cell count)

^c^ High (male: > 0.95, female > 0.85); Normal (male: < 0.95, female < 0.85)

### Risk factors for stroke at multivariate analysis

At multivariate analysis, HIV serology (OR 3.72, 95% CI 1.16–10.96), waist to hip ratio (OR 11.26 95% CI 1.98–68.24) and sickle cell disease OR 4.78 95% CI 1.11–19.70) were independent risk factors for stroke in young adults. Table 8 shows risk factors at multivariate analysis. None of the patients with HIV met the definition of AIDS as defined by the occurrence of any of the more than 20 life-threatening cancers or “opportunistic infections”, by WHO.

**Table 8:** Risk factors for stroke at multivariate analysis

	**Cases (stroke) *N*=51**	**Controls (*N*=51)**	**Adjusted odds ratio**^b^ **95% CI**	***P* values**
**HIV serology**				
Non−reactive	33 (64.7)	41 (80.4)	Reference	0.025
Reactive	18 (35.3)	10 (19.6)	3.72 (1.18–11.75)	
**waist to hip ratio**^a^				
Normal	37 (72.6)	48 (94.1)	Reference	
High	14 (27.5)	3 (5.9)	11.26 (1.64–77.24)	0.014
**Hb electrophoresis**				
AA	39 (76.5)	48 (94.1)	Reference	0.034
SS	12 (23.5)	3 (5.9)	4.78 (1.12–20.37)	
**History of family diabetes**				
Yes	4 (7.8)	8 (15.7)	Reference	0.047
No	47 (92.2)	43 (84.3)	8.48 (1.03–70.11)	
**Religion**				
Protestant	23 (45.1)	14 (27.5)	Reference	
Catholic	14 (27.5)	17 (33.3)	0.44 (0.13–1.51)	0.194
Moslem	10 (19.6)	7 (13.7)	0.53 (0.13–2.23)	0.390
Other	4 (7.8)	13 (25.5)	0.09 (0.01–0.56)	0.010
**Status of fasting blood sugar**				
Normal	44(88.0)	50(98.0)	Reference	
High	6(12.0)	1(2.0)	8.06(0.43–152.78)	0.164

^a^ High (male: > 0.95, female > 0.85); Normal (male: < 0.95, female < 0.85)

^b^ Obtained accounting for matching by age and sex using a conditional logistic regression

Variables with *p* value < 0.2 included in multivariant analysis include fasting blood sugar, hypertension, family of diabetes mellitus, waist to hip ratio, leucocyte count, HIV serology, sickle cell disease and oral contraceptive use

## Discussion

This case–control study showed that the frequency of ischemic stroke was higher than that of hemorrhagic stroke in young Ugandan population. We showed that positive HIV serology, elevated waist to hip ratio and sickle cell disease were independent risk factors for stroke in this population.

This is consistent with several studies that have been done and found ischemic stroke to be more prevalent than hemorrhagic stroke. Studies done in Africa, in Libya reported 77% ischemic stroke and 23% hemorrhagic stroke (these included both intracerebral and subarachnoid hemorrhagic stroke) [14], in Morocco, 87.3% ischemic stroke and 12.7% hemorrhagic (study did not specify on the subtypes of hemorrhagic stroke) [6]. In a study from Bosnia and Herzegovina, Subarachnoid hemorrhage was more frequent in young adults compared with older patients (> 45 years of age) (22% vs. 3.5%), intracerebral hemorrhage (ICH) was similar in both groups (16.9% vs. 15.8%), but ischemic stroke (IS) was predominant stroke type in the older group (61% vs. 74%) [15]. On the other hand, studies focusing on all young stroke patients and including also subarachnoid hemorrhages have found much higher proportion of hemorrhagic strokes in younger vs. older individuals. Population-based studies have reported as low as 57% prevalence for ischemic stroke in those aged > 45, as reported by a recent narrative review [16]. This difference in occurrence of stroke subtypes could be due to the low prevalence of hypertension in this population in our setting given that hypertension has been reported to be the commonest risk factor for hemorrhagic stroke.

Most previous studies of HIV and stroke have been retrospective, but the prospective studies in Africa and East Africa have reported the importance of HIV as a risk factor for stroke [17]. A recently published study done in Malawi, with defined cases and population controls and 99% ascertainment of HIV status, reported HIV infection as an independent risk factor for stroke. This study further found that patients who had started standard HIV treatment in the previous six months had a higher risk of stroke (OR 15.6 95% CI 4.21–46.6). This was probably due to an immune reconstitution inflammatory syndrome (IRIS) like process [18]. A variety of mechanisms have been implicated in the association of HIV and stroke, these include HIV associated vasculopathy, vasculitis which causes abnormality of the intracranial or extracranial cerebral blood vessels and neoplastic involvement. Indirectly through cardioembolic, coagulopathy in association with protein C and protein S deficiency. Some infections are well established causes of stroke, such as Mycobacterium tuberculosi*s*, syphilis, and varicella zoster virus through increased susceptibility to acquisition or reactivation of these infections [19, 20]. Combined antiretroviral therapy (cART) might unmask occult opportunistic infections that subsequently cause a stroke. This possibility should be considered in all patients who have had an acute stroke or have worsening of stroke symptoms after initiation of cART [21].

An elevated waist to hip ratio (WHR) was associated with 12 times increased risk of stroke among young adults in Mulago hospital compared to individuals with a normal waist to hip ratio. Abdominal obesity (measured as waist–hip ratio) is associated with an increased risk of myocardial infarction, stroke, and premature death [22]. This agrees with a few studies that have assessed the association of stroke with waist to hip ratio. Aaron et al. 1990, assessed the relation between body fat distribution, and the 2-year incidences of hypertension and stroke in a cohort of 41,837 women aged 55–69 years. Women who developed stroke were 2.1 (95% CI 1.5–2.9) times more likely to have an elevated ratio than those who did not [23]. Md Habib et al. 2011 assessed high waist to hip ratio as a risk factor for ischemic stroke for overall stroke and he found 64% of the ischemic stroke patient had abnormal WHR in Bangladesh [24]. Abdominal obesity measured with WHR was an independent risk factor for cryptogenic ischemic stroke (CIS) in young adults after rigorous adjustment for concomitant risk factors in the Revealing the Etiology, Triggers, and Outcome (SECRETO; NCT01934725) study, a prospective case–control study that included patients aged 18–49 years with a first ever CIS at 19 European university centers [25].

Sickle cell disease was also associated with increased risk of stroke among young adults in Mulago hospital. This agrees with several studies that have associated sickle cell disease with stroke. Ohene et al. 1998 assessed cerebrovascular accidents (CVA) in sickle cell disease, found the highest rates of prevalence of 4.01% and incidence of 0.61 per 100 patient-years. The incidence of hemorrhagic stroke was highest among patients aged 20 to 29 years [26].

In our study, the unadjusted OR for oral contraceptive use was 0.26 95% CI 0.08–0.87 with a *p* value of 0.028. This observation at the unadjusted level is significant but could be due to another variable which is a confounder to OC use such as higher socioeconomic status and better control of other possible risk factors.

In our study, we found no association between hypertension and stroke in young adults though it’s an independent risk factor for stroke in the older population. This finding is different from the multinational interstroke study which attributed most strokes among young adults in low- and middle-income countries to hypertension. In that study, only one fifth of the patients were from wealthier African countries where hypertension, diabetes and hypercholesterolemia are likely to occur with higher prevalence than in Mulago hospital [27]. Other studies have also reported the role of hypertension as a risk factor for stroke in young adults, low physical activity and hypertension were the most important risk factors, accounting for 59.7% and 27.1% of all strokes, respectively among a German nationwide case–control study based on patients enrolled in the SIFAP1 study (Stroke in Young Fabry Patients) 2007 to 2010 and controls from the population-based GEDA study (German Health Update) 2009 to 2010 [28]. A study that used population-based controls for hospitalized young patients with ischemic stroke demonstrated that independent risk factors for stroke were atrial fibrillation (OR 10.43; cardiovascular disease (OR, 8.01; type 1 diabetes mellitus (OR, 6.72; type 2 diabetes mellitus (OR, 2.31, low high‐density lipoprotein cholesterol (OR, 1.81; current smoking status (OR, 1.81; hypertension (OR, 1.43, and a family history of stroke (OR, 1.37) [29].

This finding could be explained by the high prevalence of hypertension in the general peri urban Ugandan population among young adults as reported by Kayima et al. 2015. He found a prevalence of 15% (95% CI 14.2 – 19.6%) % for young adults aged 18–44 years [30].

The study was conducted at Mulago hospital which is a national referral hospital in Uganda situated in central Uganda. Mulago hospital received patients both referred patients from all over Uganda and those from its catchment area. This is generally representative of the whole Ugandan population.

Uganda has a young population and with an HIV prevalence comparable to most countries in Sub-Saharan Africa, so the findings of this study are generalizable to other Sub-Saharan African populations.

## Conclusion

Ischemic stroke is more prevalent than hemorrhagic stroke among young adults in Mulago hospital. Independent risk factors for stroke among young adults in Mulago hospital were HIV infection, elevated waist to hip ratio and sickle cell disease. Oral contraceptive use was found to be protective of stroke among young adults in Mulago hospital. There was no significant association between stroke among young adults and hypertension, diabetes, hyperlipidemia, smoking, alcohol use and illicit use.

### Study limitations

1. The sample size was too small to detect all but the strongest associations with common exposures. When designing the study, we based on hypertension as a significant driver for strokes in this population based on other studies done to calculate the sample size, however based on our findings, hypertension was not a big driver of stroke in this population. Secondly the nature of stroke type associated with hypertension is hemorrhagic which were less common in this study. This was an unexpected finding and needs more evaluation.
2. Consecutive sampling methods has selection bias in which a variable that is associated with the outcome under investigation may occur more frequently or less in those sampled in this period as compared to the general population.
3. The use of a combined ischemic stroke and intracerebral hemorrhage group may have obscured relationships specific to one group, i.e., the risk factors for stroke were not stratified for type of stroke.
4. The best alternative for controls would have been healthy controls from the neighborhoods of the patients with stroke, but this would have been resource consuming hence the choice of hospital controls with different medical conditions from cases.

## Data Availability

The datasets used and/or analyzed during the current study are available from the corresponding author on reasonable request.
